# Comparison of Efficacy and Safety of Lispro and Aspart Evaluated by Continuous Glucose Monitoring System in Patients with Newly Diagnosed Type 2 Diabetes

**DOI:** 10.1155/2018/2087960

**Published:** 2018-03-26

**Authors:** Bing-li Liu, Guo-ping Yin, Feng-fei Li, Yun Hu, Jin-dan Wu, Mao-yuan Chen, Lei Ye, Xiao-fei Su, Jian-hua Ma

**Affiliations:** ^1^Department of Endocrinology, Nanjing First Hospital, Nanjing Medical University, Nanjing, China; ^2^National Heart Centre Singapore, National Heart Research Institute Singapore, Singapore

## Abstract

**Objective:**

To compare the effect of the rapid-acting insulin analogues (RAIAs) aspart (NovoRapid) and lispro (Prandilin) on glycemic variations by continuous glucose monitoring system (CGMS) in patients within newly diagnosed type 2 diabetes mellitus (T2DM) receiving continuous subcutaneous insulin infusion (CSII) and metformin intensive therapy.

**Methods:**

This is a single-blind randomized controlled trial. A total of 110 patients with newly diagnosed T2DM and with hemoglobin A1c (HbA1c%) above 9% was hospitalized and randomly divided into two groups: group Asp (NovoRapid group) and group Lis (Prandilin group). They all received CSII and metformin therapy. Treatments were maintained for 2-3 weeks after the glycaemic target was reached. C-peptide and insulin and fructosamine were determined. CGMS was continuously applied for 4 days after reaching the glycemic target.

**Results:**

There were no significant differences in daily dosages of insulin, fasting plasma C-P and 2 h postprandial C-P and insulin, and fructosamine at the baseline and endpoint between the groups Asp and Lis. No significant differences were seen in the 24 h mean amplitude of glycemic excursions (MAGE), 24 h mean blood glucose (MBG), the standard deviation of the MBG (SDBG), fasting blood glucose, number of glycemic excursion (NGE), and the incidence of hypoglycemia between the two groups. Similarly, no significant differences were found in areas under the curve (AUC) of glucose above 10.0 mmol/L or the decremental area over the curve (AOC) of glucose below 3.9 mmol/L between the two groups.

**Conclusions:**

Lispro and aspart had the similar ability to control the glycemic variations in patients with newly diagnosed T2DM. This study was registered with ClinicalTrials.gov, number ChiCTR-IPR-17010338.

## 1. Introduction

The continuous subcutaneous insulin infusion (CSII) therapy more closely mimics physiological insulin release with short-acting insulin analogues. Insulin lispro or insulin aspart, as the common rapidly absorbed insulin analogues, offers an advantage over regular human insulin used in insulin pumps to achieve better glucose control and quality of life. Most studies tested the effect of insulin lispro versus regular insulin [1–4]. Few clinical trials compared the efficacy and safety of lispro with aspart in type 2 diabetes mellitus (T2DM) [5, 6]. It was showed that insulin lispro or insulin aspart may have different pharmacological mechanism [7–9]. Compared with insulin aspart, subcutaneous injection of lispro has a faster absorption rate, the earlier plasma insulin peak time, and the faster rate of decline [7]. Some studies showed that insulin aspart has better chemical and physical stability than insulin lispro in insulin pumps [10]. Currently, the results on the effective and safety of insulin lispro compared with insulin aspart in type 1 diabetes mellitus (T1DM) remain controversial [7, 10–12]. Few studies have investigated the value and safety of insulin lispro compared with insulin aspart in patients with newly diagnosed T2DM by continuous glucose monitoring system (CGMS) in the Chinese population. Metformin was recommended for the treatment of type 2 diabetes as a first-line medication by the American Diabetes Association (ADA) and Chinese Diabetes Society (CDS) [13]. Recently, we reported that newly diagnosed T2DM patients who are receiving CSII therapy achieved glycemic control monitored in terms to significant improvement in the 24 h mean amplitude of glycemic excursions (MAGE) compared to those with multiple daily injections (MDI) therapy [14]. Using CGMS, we further identified that young-onset type 2 diabetes, defined as age less than 40-yrs old, received a metformin combination with CSII therapy which required significantly lower insulin doses to maintain glycemic control compared to the late onset diabetes patients [15]. Thus, the purpose of this study is to compare the efficacy and safety of insulin lispro with insulin aspart by CGMS in patients with newly diagnosed T2DM and with HbA1c more than 9%.

## 2. Methods

### 2.1. Subjects

This is a single-blind randomized controlled trial and lasted for 2-3 weeks. The study protocol and informed consent document were approved by the Institutional Ethics Committee, Nanjing First Hospital, Nanjing, China. All patients gave written informed consent. 110 patients with newly diagnosed T2DM and with HbAlc ≥ 9.0% were enrolled between February 2015 and May 2016, from Nanjing First Hospital, Nanjing, China. Patients were excluded if they were positive for antiglutamic acid decarboxylase antibodies, had severely impaired liver and kidney function and psychiatric disorders, or were pregnant or planning to become pregnant. Patients with maturity-onset diabetes in youth and mitochondria diabetes mellitus, with cognitive disorder, or abuse of alcohol or drugs were also excluded [16].

### 2.2. Study Design

All patients were admitted to the hospital. Three days after euglycemic control, fasting and 2 h postprandial blood samples were collected for measuring blood glucose, C-peptide, insulin, HbA1c%, and fructosamine in all patients before and after treatment. Then, subjects underwent oral glucose tolerance tests (OGTTs) using 75 g of glucose (dissolved in 200 ml water) [17]. CGMS data were obtained with Medtronic Minimed CGMS Gold (Medtronic Incorporated, Northridge, California, USA) for 3 days after 3 euglycemic control. All subjects received the same energy intake during the CGM periods. All patients were instructed to maintain their usual physical activity. The received meals for patients consisted of a total daily caloric intake of 25 kcal/kg/day, and the ratio of carbohydrate, proteins, and fats was 55%, 17%, and 28%, respectively.

After completing OGTTS, enrolled subjects (new diagnosed T2DM) were randomly assigned into two groups: group Asp (Novo Nordisk, Bagsvaerd, Denmark) and group Lis (Gan & Lee Pharmaceuticals, Beijing, China) in CSII, combined with metformin (Bristol-Myers Squibb, USA) therapy. During insulin intensive therapy, every patient generally uses 1.5 g metformin per day. If the patient is unable to tolerate the side effects of metformin, such as diarrhea, nausea, vomiting, and allergies, the daily dose of metformin is reduced to 1.0 g. If the patients are still unable to tolerate the daily 1.0 g of metformin, patient will be excluded from this study. Computer-generated random order was prepared by the data-coordinating center and distributed to each patient to determine the patient's treatment assignments.

The total daily dose of insulin was calculated in international units (IU). Intensive insulin treatment by CSII was initiated with an insulin pump (H-Tron Plus V100; Disetronic Medical System, Burgdorf, Switzerland). Starting insulin doses were 0.1–0.3 IU/kg, with 50% provided as bolus (premeal) injection and 50% provided as basal injection. Insulin doses were subsequently adjusted according to blood glucose values obtained by self-monitoring. The finger point blood glucose was monitored at 7 time-points: 0700, 0900, 1100, 1300, 1700, 1900, and 2200. Insulin doses were then titrated on an individual-patient basis using the algorithm (if the fasting blood glucose (0700) level was less than 4.4 mmol/L, the nocturnal basal insulin dose was reduced 0.2 units per hour from 1900 to 0600 by slowing the infusion speed; if the fasting blood glucose level was within 4.4 to 7.0 mmol/L, the nocturnal basal insulin dose would be unchanged; if the fasting blood glucose level was from 7.0 to 7.8 and 7.9 to 10.0 and >10.0 mmol/L, the nocturnal basal insulin dose would be increased subsequently by 0.2, 0.4, and 0.6 units per hour from 1900 to 0600 by increasing the infusion speed, resp.). If the prelunch blood glucose level (1100) was less than 4.4 mmol/L, the forenoon basal insulin dose was reduced 0.2 units per hour from 0600 to 1100 by slowing the infusion speed; if the prelunch blood glucose level (1100) was within 4.4 to 7.0 mmol/L, the forenoon basal insulin dose would be unchanged; if the prelunch blood glucose level was from 7.0 to 7.8 and 7.9 to 10.0 and >10.0 mmol/L, the forenoon basal insulin dose would be increased subsequently by 0.2, 0.4, and 0.6 units per hour from 0600 to 1100 by increasing the infusion speed, respectively. If the predinner blood glucose level (1700) was less than 4.4 mmol/L, the afternoon basal insulin dose was reduced 0.2 units per hour from 1100 to 1900 by slowing the infusion speed; if the predinner blood glucose level (1700) was within 4.4 to 7.0 mmol/L, the afternoon basal insulin dose would be unchanged; if the predinner blood glucose level was from 7.0 to 7.8 and 7.9 to 10.0 and >10.0 mmol/L, the afternoon basal insulin dose would be increased subsequently by 0.2, 0.4, and 0.6 units per hour from 1100 to 1900 by increasing the infusion speed, respectively [15]. Premeal insulin was divided evenly into units and given premeal. Premeal insulin doses were adjusted according to 2 h postprandial glucose levels (0900, 1300, and 1900) to achieve the target of glucose ≤10.0 mmol/L. The investigator may also terminate the adjustment of insulin dose to improve glycaemic control according to investigator's discretion and the changes should be documented in the CRF (case report form). Treatments were maintained for 1-2 weeks. The 3-day CGMS was performed after 3 days when the glycemic control with short-term intensive insulin therapy added on metformin. Glycemic control would be considered as achieved if the fasting capillary blood glucose was less than 7.0 mmol/L and 2 h postprandial blood glucose was less than 10.0 mmol/L [16, 18], meanwhile the insulin doses were unchanged during the 3-day CGMS. No antidiabetic agents were used during the intensive insulin therapy period other than metformin.

The primary endpoint was the difference of the 24 h mean amplitude of glycemic excursions (MAGE). Secondary endpoints were 24 h mean blood glucose (MBG), the standard deviation of the MBG (SDBG), number of glycemic excursion (NGE), the percentage time duration (%) of hyperglycemia (glucose > 10.0 mmol/L) and hypoglycemia (glucose < 3.9 mmol/L), the incremental area under curve (AUC) of blood glucose above 10.0 mmol/L, the decremental area over the curve (AOC) of blood glucose below 3.9 mmol/L, and hypoglycemia episodes and the effect of different interventions on insulin dose and *β*-cell function in these patients, between the two groups. Symptomatic hypoglycemic episodes and symptom-free hypoglycemia (detected with CGMS of glucose < 3.9 mmol/L) were recorded. The *β*-cell function and insulin resistance were assessed by the homoeostasis model assessment-B (HOMA-B) and HOMA-IR, calculated as previously described [16, 19]. The incremental area under curve (AUC) of blood glucose, insulin, and C-peptide were calculated using trapezoidal estimation.

Insulin and HbA1c were measured centrally at the Department of Endocrinology, Nanjing First Hospital, Nanjing Medical University. Plasma glucose was measured using the glucose oxidase method. Insulin and C-peptide were measured by chemiluminescent immunometric assay on the Modular Analytics E170 (Roche® Diagnostics GmbH, Mannheim, Germany). HbA1c was measured by high-performance liquid chromatography (HPLC) assay (Bio-Rad Laboratories Inc., California, USA). Fructosamine was determined by Glamour 2000 automatic biochemical analyzer (MD Inc., California, USA).

### 2.3. Statistical Analysis

Data were analyzed with the SPSS PASW Statistics 18 Package. For normally distributed data, the means (± standard error (SE)) of the two groups were compared using *t*-test. The rates between two groups were compared using the chi-square test. A two-way ANOVA was used in comparing the hourly glucose concentrations between the two groups. A *P* value < 0.05 was considered as statistically significant.

## 3. Results

### 3.1. Characteristics of Patients

Eleven patients were excluded from this study: 4 were from the NovoRapid group (group Asp) and 7 from Prandilin group (group Lis). The excluded eleven patients either were unable to tolerate the 1000 mg/day dose of metformin or did not reach the glycaemic control target. Thus, there were 51 patients with newly diagnosed T2DM (36 men and 15 women, mean age 51.08 ± 1.59 years, body mass index 25.78 ± 0.41 kg/m^2^) in the group Asp and 48 (37 men and 11 women, mean age 48.94 ± 1.68 years, body mass index 24.82 ± 0.54 kg/m^2^) in the group Lis. There are no significant differences in the gender composition ratio, BMI, and age between the two groups (Table 1). A total of 9 subjects were using 1000 mg/day metformin in the group Asp (5) and group Lis (4). There were no statistically significant differences in the mean dose of metformin between the group Asp (1458.33 ± 139.66 mg/day) and the group Lis (1450.98 ± 150.16 mg/day).

### 3.2. The Effect of Transient Insulin Intensive Therapy with Metformin on Glycemic Control and *β*-Cell Function

Oral glucose tolerance test was performed and the function of islet function, HbA1c, fructosamine, fasting plasma glucose, 2 h postprandial glucose, fasting plasma C-peptide, 2 h postprandial C-peptide, fasting plasma insulin, and 2 h postprandial insulin were measured before and after intensive treatment with insulin pump combined with metformin. These indices were similar between the two groups (Table 1). The HbA1c decreased by 8.14% and in group Asp versus 8.50% in group Lis, while fructosamine decreased by 21.56% in group Asp versus 20.66% in group Lis from baseline to endpoint. The differences in the reduction of HbA1c and fructosamine levels were not statistically significant between the two groups. The insulin doses were almost the same in the two groups after blood glucose target was reached. Between the group Asp and group Lis, the total insulin doses (0.26 ± 0.02 versus 0.29 ± 0.02 IU/kg), the premeal insulin dose (0.11 ± 0.01 versus 0.12 ± 0.01 IU/kg), the basal insulin dose (0.16 ± 0.01 versus 0.18 ± 0.01 IU/kg), and the intensive treatment days (10.04 ± 0.22 versus9.45 ± 0.31 days) found no significant differences. There were no statistically significant differences in the dose of metformin between the two groups.

### 3.3. The Effect of Transient Insulin Intensive Therapy with Metformin on Control of Blood Glucose Fluctuation

**Figure 1:**
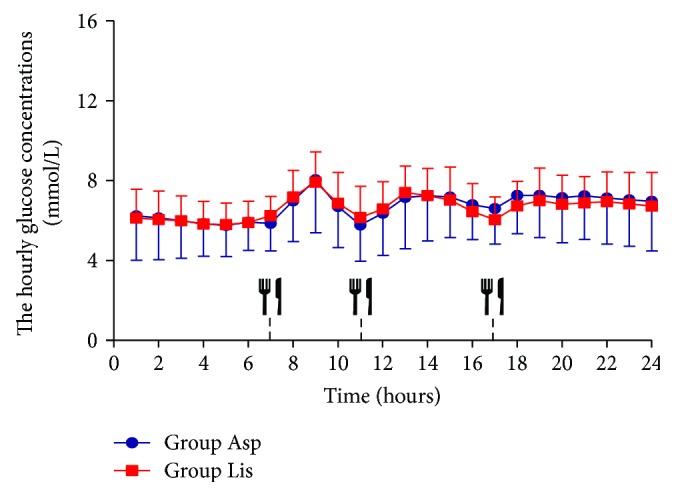
The glucose concentration per hour for 24 h by CGMS in group Asp and group Lis. The hourly glucose concentrations calculated from CGM. The hourly glucose concentrations in group Asp and group Lis. Data are presented as means ± SD. A two-way ANOVA was used in the comparison between groups. Participants were provided a standard breakfast (07:00 h), lunch (11:00 h), and dinner (17:00 h), to eat throughout the 3-day testing period.

**Figure 2:**
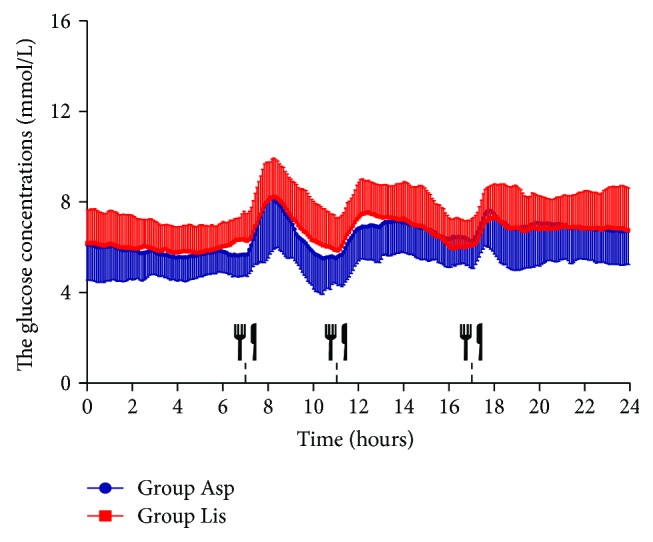
The glucose concentration per 5 minutes for 24 h by CGMS in group Asp and group Lis. The glucose concentrations per 5 minutes for 24 h from CGM between group Asp and group Lis. Data are presented as means ± SD. A two-way ANOVA was used in the comparison between groups. Participants were provided a standard breakfast (07:00 h), lunch (11:00 h), and dinner (17:00 h), to eat throughout the 3-day testing period.

We collected CGMS data at least 3 days on 2 days after glycemic control treatment by insulin pump combined with metformin. Since the continuous blood glucose monitoring data of the second day was relatively stable, we selected 24 h continuous blood glucose data of the second day to further analyze the fluctuation of blood glucose. When the patients reached the glycaemic target, the 24 h mean glucose concentration showed no significant differences between the group Asp and group Lis (6.49 ± 0.10 versus 6.49 ± 0.15 mmol/L *P* > 0.05, Table 2). The glucose concentration per hour for 24 h was similar between the group Asp and group Lis (*P* > 0.05, Figure 1). The glucose concentration per 5 minutes for 24 h was mostly similar to the group Asp and group Lis, with the exception of before breakfast (from 0630 to 0710) (Figure 2). The glucose levels before breakfast in the aspart group were transiently lower than those in the lispro group from 0630 to 0710 (*P* < 0.05), and after 07:10, there were no significant difference of glucose levels between both groups. The prebreakfast insulin doses were similar between the group Asp and group Lis (4.27 ± 0.25 versus 4.53 ± 0.44 IU, *P* = 0.608). We calculated the mean glucose before breakfast (from 0630 to 0700, 5.66 ± 0.12 versus 6.33 ± 0.15 mmol/L, *P* < 0.05) as the premeal glucose and the peak postprandial glucose after breakfast (from 0700 to 0900, 8.84 ± 0.33 versus 8.96 ± 0.24 mmol/L, *P* > 0.05) in the group Asp and group Lis. The spikes following breakfast calculated as the difference between the premeal glucose and the peak postprandial glucose found no significant differences between the group Asp and group Lis (3.18 ± 0.34 versus 2.66 ± 0.22 mmol/L, *P* > 0.05). The postprandial 2 h incremental area under the curve of the breakfast meal using the trapezoidal method found no significant differences between the group Asp and group Lis (198.78 ± 27.27 versus 142.08 ± 128.98 mmol/L × 120 min, *P* = 0.095).

The MAGE in group Asp had no significant differences as compared with group Lis (group Asp 3.33 ± 0.27 mmol/L, group Lis 3.18 ± 0.19 mmol/L). There was no significant difference of NGE and SDBG between the two groups (Table 2). We calculated the AUC and the time spent in 10.0 mmol/L and the AOC and the time spent in 3.9 mmol/L as the cut-off point in the two groups (Table 2). The incremental AUC (>10 mmol/L) detected by CGMS did not significantly decrease (0.04 ± 0.01 mmol/L per day) in group Asp as compared with group Lis (0.04 ± 0.01 mmol/L per day). The time in normal glycemia (between 3.9 and 10.0 mmol/L) in group Asp did not significantly increase compared to group Lis (97.59 ± 2.48% in group Asp versus 94.81 ± 1.05% in group Lis).

### 3.4. The Hypoglycemic Episodes in Group Asp versus Group Lis

No severe hypoglycemic episodes, defined as an event requiring the assistance of another person or other resuscitative treatments, were reported in any treatment group. The number of hypoglycemia events (<3.9 mmol/L) in group Asp was 22 and it was 15 in group Lis. There were no statistical differences found between the two groups by chi-square analysis (chi square = 1.493, *P* = 0.222). The decline AOC and the time when blood glucose < 3.9 mmol/L as detected by CGMS had no significant differences between the two groups.

## 4. Discussion

In the present study, CGMS data showed that lispro had a similar effect as aspart on blood glucose fluctuation and hypoglycemia. There were no significant differences seen in MAGE, 24 h MBG, SDBG, fasting blood glucose, and NGE between two groups in patients with new diagnosis of T2DM. The results indicated that the blood glucose fluctuation were similar in group aspart and group lispro. Meanwhile, the AOC of 3.9 mmol/L as the cut-off point was similar between the two groups, suggesting that the patients with newly diagnosed T2DM would have the similar risk of hypoglycemia when using aspart or lispro.

The rapid-acting insulin analogues (RAIAs) aspart and lispro have been approved for use in CSII therapy in patients with diabetes. Some studies have demonstrated that aspart has greater compatibility for use in insulin pumps in comparison with lispro or glulisine [13]. Studies demonstrate that aspart has a more rapid rate of absorption [20–24], more stable postprandial control [10, 11, 25–27], and lower rates of fibrillation and occlusion [10, 28–31] for use in insulin pumps compared with lispro. These qualities make aspart compatible for use in CSII therapy and perhaps a better choice of insulin to ensure improved outcomes, which in turn may result in improved treatment adherence. Meanwhile, the study by Tamborlane et al. showed lispro had a decreased rate of hypoglycemia compared with aspart in type 1 diabetes [12]. Our data was partially agreed with the study reported in CSII in adult patients with noninitial diagnosis of T2DM [6]. Insulin lispro was not inferior to insulin aspart in HbAlc, total daily insulin dose, weight change, and incidence and rates of hypoglycemia [6]. In that study, the patients operated the insulin pumps by themselves at home, which may cause operation errors. Our study selected patients with newly diagnosed T2DM, and insulin pump and CGM were uniformly operated by trained nurses to avoid the operation errors. The patients' diet had a unified diet in the hospital to avoid interference by casual dining outside the hospital. We found that there were no significant differences in daily dosages of insulin, C-P 0, C-P 2 h, Ins 0, Ins 2 h, HbA1c, and fructosamine between group Asp and group Lis before and after treatment. The glucose concentration per hour for 24 h was similar between the group Asp and group Lis, but the glucose concentration per 5 minutes had transient differences from 0630 to 0710 (before breakfast). These differences may be related to the moderate sample size. The spikes following breakfast and the postprandial 2 h incremental area under the curve of the breakfast meal found no statistical differences between the group Asp and group Lis. These data suggested that the effects of the two kinds of insulin were similar in reducing postprandial glucose for breakfast.

More currently, clinical trial results, comparing the effectiveness and safety of insulin lispro and insulin aspart, were controversial in patients with T1DM [12]. Thus, the aim of the current study is to investigate the efficacy and safety of aspart and lispro delivery by insulin pump combined with metformin using CGMS in patients with newly diagnosed T2DM. Compared with the conventional 7-point peripheral blood glucose monitoring, CGMS can record the blood glucose automatically every 5 minutes. CGMS can monitor the dynamic changes of blood glucose within 72 hours to show the daily change of blood glucose curve accurately [16]. Our present CGMS data showed no statistical significances in 24 h MBG (6.49 ± 0.10 versus 6.49 ± 0.15 mmol/L), the glucose concentration per hour, MAGE (3.33 ± 0.27 versus 3.18 ± 0.19 mmol/L), SDBG (1.30 ± 0.08 versus 1.28 ± 0.06 mmol/L), NGE (4.54 ± 0.27 versus 4.60 ± 0.21 times), and 10.0 mmol/L as the cut-off point calculation with the blood glucose area under the curve (0.04 ± 0.01 versus 0.04 ± 0.01 mmol/L per day) and the duration of blood glucose (3.00 ± 0.86 versus 3.00 ± 0.78%) between the group Asp and group Lis in patients with newly diagnosed T2DM. The data showed that the two RAIAs had the similar function in controlling the level of blood glucose and blood glucose fluctuation.

The current study also analyzed the hypoglycemia episode in the two groups. There was no severe hypoglycemia event and symptomatic hypoglycemia found during the study period between the two groups. The number of blood glucose less than 3.9 mmol/L and the AOC of glucose below 3.9 mmol/L was similar between the two groups. The data indicated that aspart and lispro had no difference in hypoglycemic events.

In summary, aspart and lispro had similar effects on blood glucose fluctuation, 24 h average blood glucose, fasting blood glucose, and the incidence of hypoglycemia in new diagnosis of T2DM. Because of the considerable effect of aspart and lispro, they can both be regarded as suitable insulin intensive treatment options.

## Figures and Tables

**Table 1:** The characteristics of patients and the effect of transient insulin intensive therapy with metformin on glycemic control and *β*-cell function.

Group	Group Asp (51)	Group Lis (48)	*t* value	*P* value
Age (years)	51.08 ± 1.59	48.94 ± 1.68	0.927	0.356
BMI (kg/m^2^)	25.78 ± 0.41	24.82 ± 0.54	1.450	0.150
Weight (kg)	71.00 ± 12.18	74.80 ± 10.75	−1.630	0.106
The insulin does/day per kilo of weight (U/kg)	0.26 ± 0.02	0.29 ± 0.02	−0.632	0.529
The intensive treatment days (days)	10.04 ± 0.22	9.45 ± 0.31	1.581	0.117
Before intensive treatment				
HbA1c (%)	10.71 ± 0.20	10.99 ± 0.23	−0.909	0.366
Fructosamine (*μ*mol/L)	412.62 ± 12.53	427.65 ± 14.97	−0.775	0.44
Fasting plasma glucose (mmol/L)	10.65 ± 0.35	11.31 ± 0.44	−1.182	0.239
2 h postprandial glucose (mmol/L)	21.73 ± 0.57	21.24 ± 0.65	0.561	0.575
Fasting plasma C-peptide (pmol/L)	2.20 ± 0.13	3.23 ± 0.84	−1.254	0.212
2 h postprandial C-peptide (pmol/L)	4.46 ± 0.24	5.01 ± 0.38	−1.250	0.214
Fasting plasma insulin (mU/L)	7.71 ± 0.82	8.48 ± 0.64	−0.737	0.462
2 h postprandial insulin (mU/L)	19.75 ± 1.80	25.36 ± 3.29	−1.511	0.133
HOMA-IR	3.75 ± 0.42	4.26 ± 0.36	−0.927	0.356
HOMA-B	24.50 ± 3.00	26.41 ± 3.41	−0.421	0.674
After intensive treatment				
HbA1c (%)	9.35 ± 0.18	9.24 ± 0.25	0.337	0.737
Fructosamine (*μ*mol/L)	330.13 ± 7.42	322.51 ± 10.44	0.609	0.544
Fasting plasma glucose (mmol/L)	6.90 ± 0.26	6.81 ± 0.24	0.254	0.799
2 h postprandial glucose (mmol/L)	16.29 ± 0.49	15.38 ± 0.54	1.261	0.21
Fasting plasma C-peptide (pmol/L)	2.10 ± 0.16	2.21 ± 0.10	−0.591	0.555
2 h postprandial C-peptide (pmol/L)	8.63 ± 1.50	7.38 ± 0.43	0.785	0.434
Fasting plasma insulin (mU/L)	6.03 ± 0.50	6.99 ± 0.70	−1.131	0.26
2 h postprandial insulin (mU/L)	40.01 ± 3.97	40.15 ± 5.11	−0.022	0.981
HOMA-IR	1.86 ± 0.17	2.18 ± 0.27	−1.014	0.312
HOMA-B	42.73 ± 5.00	51.46 ± 6.52	−1.071	0.286

Data were presented as means ± SD. BMI: body mass index; HOMA-B: homeostasis model assessment-B; HOMA-IR: homeostasis model assessment-IR.

**Table 2:** The effect of transient insulin intensive therapy with metformin on blood glucose fluctuation control compared group Asp and group Lis.

	Group Asp (51)	Group Lis (48)	*t* value	*P* value
24 h MBG (mmol/L)	6.49 ± 0.10	6.49 ± 0.15	0.032	0.975
SDBG (mmol/L)	1.30 ± 0.08	1.28 ± 0.06	0.157	0.876
MAGE (mmol/L)	3.33 ± 0.27	3.18 ± 0.19	0.703	0.484
NGE (times)	4.54 ± 0.27	4.60 ± 0.21	−0.185	0.853
>10 AUC (mmol/L/day)	0.04 ± 0.01	0.04 ± 0.01	−0.014	0.989
>10 time (%)	3.00 ± 0.86	3.00 ± 0.78	−0.142	0.887
<3.9 AOC (mmol/L/day)	0.00 ± 0.00	0.01 ± 0.00	−0.481	0.246
<3.9 time (%)	1.71 ± 0.42	2.27 ± 0.79	−0.956	0.524
≥3.9 and ≤10 AUC (mmol/L/day)	2.55 ± 0.10	2.67 ± 0.11	−0.193	0.439
≥3.9 and ≤10 time (%)	97.59 ± 2.48	94.81 ± 1.05	−0.63	0.315

Data were presented as means ± SD. MBG: mean glucose concentration (mmol/L); SDBG: the standard deviation of the MBG (mmol/L); MAGE: mean amplitude of glycemic excursions (mmol/L); NGE: number of glycemic excursion; >10 AUC: the incremental area under curve of glucose concentrations above 10.0 mmol/L (mmol/L per day); >10 time: the percentage of the time spend on glucose concentrations above 10.0 mmol/L; <3.9 AOC: the decremental area over curve of glucose concentrations below 3.9 mmol/L (mmol/L per day); <3.9 time: the percentage of the time spend on glucose concentrations below 3.9 mmol/L; ≥3.9 and ≤10 AUC: the incremental area under curve of glucose concentrations between 3.9 mmol/L and 10 mmol/L (mmol/L per day); ≥3.9 and ≤10 time: the percentage of the time spend on glucose concentrations between 3.9 mmol/L and 10 mmol/L.
